# Characteristics of work in primary care identified in the collective exercise of application of the SWOT matrix

**DOI:** 10.1590/0034-7167-2022-0443

**Published:** 2023-03-17

**Authors:** Maristela Izcak Baldissera, Carine Vendruscolo, Denise Antunes de Azambuja Zocche, Fabiane Ferraz, Rafael Gue Martini

**Affiliations:** I Universidade do Estado de Santa Catarina. Chapecó, Santa Catarina, Brazil; II Universidade do Extremo Sul Catarinense. Criciúma, Santa Catarina, Brazil

**Keywords:** Primary Health Care, Family Health, Management Quality Circles, Nursing, Work., Atención Primaria de Salud, Salud de la Familia, Participación em las Decisiones, Enfermería, Trabajo., Atenção Primária à, Saúde, Saúde da Família, Participação nas Decisões, Enfermagem, Trabalho.

## Abstract

**Objective:**

to understand how variables strengths, weaknesses, opportunities and threats make it possible to characterize work in Primary Health Care, to collectively propose strategies for systematizing this process with Family Health teams.

**Methods:**

methodological research, with 23 participants in direct relationship with Primary Care, including representatives of segments: care (work), management and social control. In one of the three pedagogical meetings, the SWOT matrix was used as a tool for organizational work planning.

**Results:**

applying the matrix resulted in the following thematic categories: Teamwork and regulations: key elements for interprofessionality at work; Continuing Health Education: path to autonomy and participatory management (co-management).

**Final considerations:**

the study promoted the systematization of teams’ work, through the mobilization of sectors and distribution of tasks, involving professionals in the co-management of the process.

## RESUMO

**Objetivo:**

compreender como as variáveis forças, fraquezas, oportunidades e ameaças possibilitam caracterizar o trabalho na Atenção Primária à Saúde, para propor coletivamente estratégias de sistematização deste processo junto às equipes de Saúde da Família.

**Métodos:**

pesquisa metodológica, com 23 participantes em relação direta com a Atenção Primária, contemplando representantes dos segmentos: atenção (trabalho), gestão e controle social. Em um dos três encontros pedagógicos, utilizou-se a matriz SWOT como ferramenta para o planejamento organizacional do trabalho.

**Resultados:**

a aplicação da matriz resultou nas seguintes categorias temáticas: *Trabalho em equipe e normativas: elementos-chave para a interprofissionalidade no trabalho; Educação permanente: caminho para a autonomia e para a gestão participativa (cogestão).*

**Considerações finais:**

o estudo promoveu a sistematização do trabalho das equipes, mediante a mobilização dos setores e a distribuição de tarefas, implicando os profissionais na cogestão do processo.

## RESUMEN

**Objetivo:**

comprender cómo las variables fortalezas, debilidades, oportunidades y amenazas permiten caracterizar el trabajo en la Atención Primaria de Salud, para proponer colectivamente estrategias para sistematizar ese proceso con los equipos de Salud de la Familia.

**Métodos:**

investigación metodológica, con 23 participantes en relación directa con la Atención Primaria, incluidos representantes de los segmentos: cuidado (trabajo), gestión y control social. En uno de los tres encuentros pedagógicos se utilizó la matriz FODA como herramienta para la planificación del trabajo organizacional.

**Resultados:**

la aplicación de la matriz resultó en las siguientes categorías temáticas: Trabajo en equipo y normativa: elementos clave para la interprofesionalidad en el trabajo; Educación Permanente en Salud: camino hacia la autonomía y la gestión participativa (cogestión).

**Consideraciones finales:**

el estudio promovió la sistematización del trabajo de los equipos, a través de la movilización de sectores y la distribución de tareas, involucrando profesionales en la cogestión del proceso.

## INTRODUCTION

In Primary Health Care (PHC), the systematization of work makes up the framework of actions for its organization in the Health Care Network (RAS - *Rede de Atenção à Saúde*), influencing local management and ordering of other services. The strengthening of PHC^,^ designated as Primary Care (PHC) in Brazil, represents one of the most significant advances of the Unified Health System (SUS - *Sistema Único de Saúde*) as a public policy aimed at universal, equitable and comprehensive health. In this regard, PHC is considered the main “gateway” to SUS, and its evolution occurred through the scope of Family Health Strategy (FHS), which fostered a change in the care model and enabled an increase in the supply of actions and services, producing results positive about the population’s health^(1)^.

Family Health teams (FHt) are made up of a physician, a nurse, a nursing assistant and/or technician and community health workers (CHW), making oral health professionals optional: dentist and auxiliary or technician in oral health and endemic combat agent (ECA). The 2000s were marked by the expansion of FHS, with the incorporation of oral health teams and the creation of the Expanded Centers for Family Health and Primary Care (NASF-AB - *Núcleos Ampliados de Saúde da Família e Atenção Básica*). Several criticisms were made of this expansion, such as inadequate infrastructure, underfunding, the care model and the lack of attraction of medical professionals by the^(2-3)^ model.

Although government proposals, expressed through policies and ordinances, have been weakening the pillars that support the SUS, since 2016, the need to strengthen the system is highlighted, based on collaborative management (co-management). This initiative is in line with cooperativeness without hierarchy between the points of care established for the RAS constitution, relating the levels and sectors responsible for the longitudinal care of people^(4)^.

The 2017 Brazilian National Primary Care Policy (PNAB - *Política Nacional de Atenção Básica*) guides the development of technical mechanisms and organizational strategies for the qualification of multidisciplinary teams, in management and care dimensions. In an unprecedented way, this version of the policy recognizes the “PC Manager” and considers, among its attributions, to identify training/qualification needs together with the team, for improvements in work, quality and resolution of care^(5)^.

In PHC, work is considered complex and sometimes presents difficulties that demand skills, in order to promote a good interpersonal relationship between team members, users’ direct access to professionals and care follow-up. With a view to assisting in this process, some management technologies show promise for team planning, such as the SWOT analysis or matrix, theoretical model applied in strategic management that aims to carry out situational analysis, taking into account the internal and external factors that permeate the service. The SWOT matrix (Strengths, Weaknesses, Opportunities and Threats) makes it possible to carry out a diagnosis of the place, exchange and share different knowledge, helping to build a strategic agenda and public governance. It aims to identify the degree to which strengths and weaknesses are significant and capable of resolving threats or capitalizing on opportunities in the work environment^(6-7)^.

From the need to identify the elements that influence the systematization of work in health services, this study sought to use the SWOT matrix as a management technology for the situational analysis of this process in PHC.

## OBJECTIVE

To understand how variables strengths, weaknesses, opportunities and threats make it possible to characterize work in PHC, to collectively propose strategies for systematizing this process with FHt.

## METHODS

### Ethical aspects

The study respected the ethical aspects regulated by the Brazilian National Health Council. It is housed in macro-research, approved by the Research Ethics Committee of the proposing institution. The consent of the institutions involved was requested as well as the signing of the Informed Consent Form by all those directly involved in the research. Participants were guaranteed information, the possibility of participating/abandoning the research and anonymity in the treatment of the information obtained, using codenames based on the letter P (Participant), followed by the initial of the segment it represents (C - Care; M - Management) and a cardinal number.

### Study design

This is methodological research with qualitative approach. This type of research consists of production/construction, validity and assessment of reliable research instruments and techniques, which can be used by other researchers, in order to elaborate a product^(8)^.

### Methodological procedures

The methodological study phases were adapted to the following steps: 1) Exploratory phase - the theme was defined and the diagnosis was built through the SWOT matrix, whose elements highlighted by participants gathered information and identified possible elements to be problematized; 2) Problematization - problems that could be modified were identified; 3) A guide was built to systematize the FHt’s work, through integrative seminars that sensitized and encouraged the group to reflect and make decisions; 4) Validity and dissemination of results - the guide was validated by judges and resumed with the group and results, which were published^(8)^.

This article presents and discusses information from phases 1 and 2, which occurred through descriptive-exploratory research strategies. The phases took place between July and August 2021, through three meetings with FHt professionals.

### Study setting and participants

The study was conducted in a municipality in western Santa Catarina using PHC. The municipality’s PHC has seven FHt, distributed in five Basic Health Units (BHU). FHS covers 100% of the population. All teams have a doctor, a nurse, a nursing technician, CHW, an oral health assistant and/or technician and a dentist.

Participants were intentionally chosen, as the proposal made it possible to apply a management technology (the SWOT matrix) that valued the daily life and demands identified by those involved in PHC work in the municipality in question. Twenty-nine professionals were invited to participate, including managers, workers and social control representatives, and 23 accepted the invitation, representing the care, management and social control segments, which work in interface with the seven PHC in the municipality. This representative proposition meets the “Health Education Prism” ideas.

The “Prisma” is a metaphor that expands the formulation that the actors who represent management, health education, workers and social control are co-responsible for professional training^(9)^. From this perspective, it should be noted that the research is based on the methodological theoretical construct of Continuing Health Education (CHE), which considers SUS as a school, i.e., practice scenarios are live scenarios of pedagogical production^(10)^. In this study, however, the social control segment had little representation, and the teaching segment was not a participant in the research, however, it was represented by the researcher.

The following inclusion criteria were adopted: 1) For the care segment - PHC professionals who had been working in the health service for at least six months; 2) Representatives of the management segment - to be acting as secretary of health (or representative appointed by him) in the municipality, or other collaborators who acted in the management team, during the period of field work; 3) Social control/user segment representative - residing in the assigned territory for more than 10 years and playing a role of representativeness and leadership in spaces of representation in health.

The 23 participants are represented as follows: in care - 10 nurses, a physician, a dentist, an CHW, a pharmacist and two multidisciplinary team representatives, former NASF-AB; in management - the secretary of health, PHC’s general director, two BHU coordinators, the PHC coordinator, the epidemiological surveillance coordinator; in social control - a Municipal Health Council representative.

### Data collection and organization

During fieldwork, group interviews were used as a methodological resource, which had a semi-structured group interview guide, which conducted the dialogue. The meetings lasted an average of two hours. There was an average participation of 20 participants and they were held at the Municipal Health Department (MHD) premises.

The co-management of health work was taken as a proposed reference for the systematization of work in PHC, because it is a democratic possibility of power sharing and for the understanding and intervention of subjects in a certain context, in this case, FHS^(4,11)^.

In this article, the results of the first and, especially, of the second meeting, in which the SWOT matrix was applied, will be analyzed and discussed. After a diagnosis, carried out in the first meeting, in which the topics to be discussed and which could contribute to the systematization of teams’ work through CHE, were defined, using the SWOT matrix was suggested to assist in addressing the identified problems, as well as mapping the skills of those involved.

In the second meeting, 23 people participated, organized into two groups. Each group worked on two matrix variables: the first, strengths and weaknesses in the internal environment, and the second, opportunities and threats in the external environment. In this meeting, the analysis of all the elements expressed in the previous meeting was carried out, aiming at fitting them into the variables and identifying key elements that influence the systematization of teams’ work in the municipality’s PHC services.

The information generated in the group interviews was recorded and later transcribed in full for analysis.

### Data analysis

For processing the data revealed in the two meetings that used the matrix, thematic content analysis^(12)^ was used following the moments: pre-analysis, material exploration and data processing. The Paideia Training and Support Methodology was adopted as a theoretical-philosophical reference, for the co-management of work and health networks, which operates from praxis as a democratic possibility of sharing power in collective spaces^(11)^. Two categories emerged: *Teamwork and regulations: key elements for interprofessionality at work; Continuing Health Education: path to autonomy and participatory management (co-management)*.

## RESULTS

### Teamwork and regulations: key elements for interprofessionality at work

In the internal environment, the strengths variable brought out elements that generated dialogue between professionals, such as the need for a team meeting, considered an important and necessary strategy. However, they observed a lack of time for it, as well as effective teamwork, with the exchange of knowledge between professional categories and with other sectors. These items were identified in the weakness variable and were attributed to the lack of organization and to factors that could qualify teamwork and collaborative action.

[...] *sometimes we don’t have the time we would like to hold our meetings, as it should be.* (PM1)

[...] *we missed Thursday, which was the day we closed the BHU, had a meeting with the team, used it to plan and catch up on some things* [...]. *It’s that rush, we don’t talk anymore and I miss that.* (PC5)

[...] *I would like to work more in a team. We did some things together, but they are more specific things, we exchange ideas, but there is a lack of a programmatic agenda involving everyone.* (PC6)

[...] *the discussion of different types of knowledge* [...] *sometimes it happens between two people, but the more collective it is, the better, the more chances of success.* (PC1)

For a possible referral on this point, PC3 makes colleagues reflect on the importance of holding meetings and strengthening as a team:

[...] *notice how the first thing we lose is this, the more overwhelmed we are, what do we take away? The space where we could organize the mess! These issues, I see that they are only for the team, there is no point in waiting for the management, we need to strengthen ourselves as a team* [...]. (PC3)

In this perspective, they again reinforce the importance of more collaborative (exchange of knowledge and collective practice) and interprofessional practices, as through therapeutic projects:

[...] *as far as possible, we seek, through exchange of information/knowledge with the team, with colleagues, to qualify the work! Also, with doctors, technicians, the knowledge they have, it is very important/very valuable to work together!* (PC2)

[...] *I consider interprofessionality and Therapeutic Projects fundamental.* (PC3)

[...] *when you take a case to work colleagues, when you start to share that problem or to seek a solution for that patient* [...] *we become more accomplices, more partners, more concerned about the outcome of work, because you can lean on your colleague.* (PM1)

Although the existence and use of norms, manuals and protocols, whether municipal, state or federal, has been signaled as an opportunity, lack of appropriation by some professionals regarding their existence and content was punctuated as a weakness, as well as the difficulty of complying with prescriptions that are not consistent with the local regional reality:

[...] *we work a lot with regulations, with things that we have to adhere to. There are things that we have difficulty with, we would like to do differently, but, according to the norm, we have to do it this way.* [...] *in the work process, everyone should do it the same way, each team does it one way, right?* (PC8)

The “performance indicator” was an element that generated discussion among participants. These indicators are part of the list of components of the new PHC funding model within the scope of SUS - “*Previne Brasil*”, established in 2019. They were identified as an opportunity by some, but also as a threatening element in PHC services, as professionals see them as an imposition/obligation of a productivist logic:

[...]*diabetes and hypertension indicators will drive the improvement of the work process, follow-up* [of patients], *but it is a threat in relation to the resource.* (PC4)

[...] *maybe, in that first moment, when they [indicators] arrived here, the way they arrived, they are more threatening than opportunities, but in the long term I think they tend to create opportunities for improvements.* (PC9)

[...] *I think their nature* [indicators] *makes no sense, this longitudinal thing, this hierarchical thing, of placing indicators from top to bottom, disregarding all the differences that exist, that is useless! If you don’t have people who know how to deal with them and make something useful out of them, it’s just a number to receive a resource* [...]. (PC6)

In the new PHC financing model, the quality of the records and the transfer of information are essential, but this information, when coming from the government, in relation to the dynamics of the program’s operation, have many edges, and this point is considered a threat. The lack of information on the allocation of resources in health was evident, identifying the failure in the communication process.

[...] *what I understand is that, initially, it was not very widespread, so we learn about the indicator when the resource has already been compromised due to non-compliance with that goal.* (PC7)

[...] *communication is a very serious problem, not only internally, but also between teams/sectors, sometimes people think they hold the knowledge, they don’t pass it on, which makes this process very difficult - and a lot -.* (PC4)

Based on these data, it can be seen that teamwork, as well as institutional regulations, can configure key elements for interprofessionality in teams’ work.

### Continuing Education: path to autonomy and participatory management (co-management)

Flagged as a highlight in the strength variable, professionals’ autonomy for the development of their activities emerges, although the manager indicates that some professionals assume a passive attitude instead of solving problems:

*When we try to give autonomy to the team, there are some who don’t want it. It is worth reflecting on how much the professional wants to have autonomy, contribute to these processes, or just complain* [...]. (PM2)

Important elements, highlighted as weaknesses, which imply the development of autonomy, are the lack of knowledge of most professionals in relation to public policies, few health promotion actions and lack of disclosure about those that exist. For the latter, dissemination through electronic media is suggested:

[...] *we work a lot and disclose little* [...] *nowadays, with channels and social networks for dissemination, we could take advantage of much more, even thinking about health promotion and prevention. Why not make a five-minute program/video to post on social networks with different themes?* (PC6)

[...] *you need to have qualified people who produce good material, something attractive, you have to understand the media.* (PC3)

On the other hand, the existence of a job and career progression policy is understood as an opportunity in which professionals can qualify, supported by the guarantee of their release from work. Although they recognize this opportunity, including for autonomy, they also make some criticisms in the sense of how the management could improve these elements, taking advantage of them as pedagogical moments for the team:

*In terms of motivation, in our assessments* [progression by merit, from the municipal plan], *professionalism is assessed, but they should be taken advantage of, in the sense of improving/transforming the work process* [...]. (PC3)

[...] *there could be assessment with peers, exchanges. Our assessments, progression based on merit, do not drive us towards transformation, I think it had to be something not so fast, so that we could use it for our professional growth.* (PC3)

When reflecting on how to forward the problems highlighted, the participants related the opportunities to structuring the Municipal Nucleus of Continuing Education in Health and Humanization (NEPSHU - *Núcleo Municipal de Educação Permanente em Saúde e Humanização*), that would represent the possibility for implementing CHE as a management strategy.

[...] *we have to have this knowledge and strengthen continuing education actions. The System* [SUS] *sees this logic of health as a commodity* [...] *we have to fight as a SUS server, to be able to make health happen!* (PC3)

Hence, the idea of creating a NCHEHU encouraged participants in relation to their empowerment, as a possible path to autonomy and, above all, to collective decision-making, i.e., to participatory management.

## DISCUSSION

Both FHt’s and Primary Care (PCt) teams’ work can (and should) be linked to the possibility of negotiating decision-making processes, with the collective and reflective construction of knowledge, respect for differences and singularities of knowledge and practice cores, through dialogic processes^(9)^. This prerogative for work in PHC emerged strongly (and in the strength variable) among participants, during the matrix SWOT application. They signaled skills that teams need to rescue communication, organization and planning in PHC, which makes them reflect on the need to develop skills of this nature in individuals who attend the same work environment^(7)^.

The systematization of work can be mediated by spaces guaranteed in team meetings, which are very important for communication and interprofessional relationships. Team meetings become significant instruments for the organization and planning of PHC professionals’ daily work^(13)^. They can occur through the discussion of cases, in an interdisciplinary way, development of CHE actions/activities and socialization of knowledge. However, it is known that changes in the regulatory processes for conducting PHC in recent years are forcing changes in the logic of action, which sometimes causes the first thing to be excluded are spaces for dialogue and collective planning, to the detriment of the need for agendas to meet the population’s needs.

This organizational structure, which encourages the use of persuasion and negotiation instruments corroborates with the Paideia methodology ideas, which does not conceive of social organization without the dispute for power and even considers this dispute essential to democracy. It presupposes the existence of shared projects from the management that make it possible to leave oneself, without, however, abandoning oneself^(6,14)^. Thus, co-management functions are exercised between subjects with different levels of knowledge and power, who deal with their affections through negotiation, mediation of conflicts and actively participate in the process^(6,14)^.

The experience of using the SWOT matrix for a collective construction of the team encouraged the exercise of collaborative practice, recognized as a potential of that group. This practice goes beyond interprofessional issues, it integrates the overview of users, families and communities in the search for caring for people instead of caring for people, with users as a central element^(15-16)^. In this perspective, it is important to recognize the specific competencies of each profession and also those collaborative, in which tolerance and negotiation are imperative^(17)^. These aspects were well evidenced in the study, whose representatives of the different segments problematized ideas present in the Interprofessional Education (IPE) theoretical framework ^(15-16)^. IPE aims to build knowledge in the collective, through dialogue and respect for the differences of a group^(15)^ with several categories and with different knowledge cores^(15-16)^.

The concept of education centered on the unidirectional transmission of rational scientific knowledge is still very present in institutions. This conception results in attitudes in which educators (and health professionals) are responsible for “passing on” updated scientific information, while students (and users) must have a passive and obedient attitude of gathering information (preventive, diagnostic and therapeutic) and reproduction of prescriptions^(9,17)^. This authoritarian figure, played by a teacher or health professional, aimed at the obedience of students (and users), reproduces authoritarian, hierarchical and controlling relationships, based on the threat and fear of punishment. This reality reinforces the need for pedagogical processes in health production scenarios and in teaching institutions that mobilize dialogue, horizontality and respect in relationships.

PHC needs professionals who expand its core of knowledge, beyond technical competence, incorporating knowledge of public policies and health work management, with a view to a better organization of work^(18)^. This lack of knowledge was highlighted as a weakness, along with the lack of professionals in PHC, which causes work overload, demotivation, physical and mental exhaustion.

Autonomy, highlighted as strength, is important in any profession, capable of increasing the degree of satisfaction/pleasure in the work performed. It implies freedom to make decisions, based on scientific evidence, based on legal provisions, such as public policies, Ministry of Health protocols, or created by municipalities, among others. In nursing, for instance, one of the strategies to promote new profiles of nurses would be to increase autonomy in the profession’s practices, to generate more advanced and more resolute care in PHC, through instrumentation and professional regulation^(19)^. In the present study, it is evident that the autonomy of professionals is a positive factor and derives mainly from clinical practice, supported by care protocols, especially in nursing.

In addition to the duties common to all PHC team professionals, those specific to nurses, prescribed by the PNAB^(5)^, include conducting consultations with nurses, requesting complementary exams, prescribing medications, - observing the legal provisions of the profession and following protocols or norms established by the Ministry of Health, state, municipal or Federal District managers -, in addition to referrals, when necessary, from users to other RAS services. In Brazil, the Law of Professional Nursing Practice guarantees nurses the right to prescribe medications, through protocols, in Nursing Consultation. PHC nurses expand autonomy through clinical practice supported by comprehensive care^(20)^.

PHC vocational activities, such as prevention and health promotion, were remembered by the participants as basic prerogatives for the systematization of work. They, in fact, strengthen the bond with users, enabling them to become the main actor in their self-care^(21)^. Profitably, participants also signaled a problem that is common in SUS network services: the lack of specialized professionals, which implies comprehensive care. In this sense, it is worth remembering that, historically, the integrality attribute represents one of the greatest critical nodes of PHC and SUS, especially for flow regulation^(22)^.

It should be remembered that in the 2000s, in order to solve the previously expressed problem, Family Health Support Centers - NASF (current NASF-AB) were created, which, although they lost funding with the new financing model (*Previne Brasil*), remain in some municipalities, as is the case of this study. By observing the regulations, NASF do not configure PHC services, as they do not operate with direct access; however, in practice, professionals work in preventive clinical actions, planning and health promotion. They can, therefore, be configured as a partly specialized team (secondary care), located at the intersection between PHC and secondary care, in addition to contributing to management of analysis of priorities (performed by FHt/PCt) and the flows (management and specialized care regulation)^(17)^.

In line with CHE and humanization policies, many problems highlighted by the participants, in variables weaknesses and threats, pass through the team’s pedagogical attitude, i.e., daily exercise of seeking solutions based on different experiences and knowledge present in the team. In the Paideia method, co-management or shared management is considered as a possibility to think about the practice and recognize the subject and their role in this context. Dialogic spaces of co-management are, therefore, loci of learning and transformation that can impact qualitative health production^(11)^.

In order to strengthen CHE’S actions, the structuring of NCHEHU in the municipality emerged as an opportunity for change. Since 2010, in compliance with the CHE Policy, the Santa Catarina State Commission for Teaching-Service Integration (State CIES - *Comissão de Integração Ensino-Serviço*) and Continuing Education Division (DEP-SC - *Divisão de Educação Permanente*) have sought to mobilize the regional CIES for the reflection and implementation of CHE municipal centers in health regions. Periodic meetings and workshops, organized by the state CIES, based on local research, expressed the importance of creating municipal or micro-regional CHE centers to strengthen the regions^(23)^.

NCHEHUs must add and strengthen the CHE and humanization policies, within the scope of municipalities or micro-regions, having their principles, guidelines and objectives aligned, with a view to strengthening the SUS, through changes in work in PHC. The intention is that the NCHEHU are a management tool at work. The first centers created in the State, in the Greater Florianópolis and Middle Vale do Itajaí regions, provoked mobilization for training and transforming actions in work processes as well as helping managers in decision-making. They are spaces for dialogue, which provide a multidisciplinary view, considering service users’ point of view, seeking consensus and commitment to the actions proposed at the local level^(23)^.

Participants in this study point out known tools for resolving PHC problems, including collaboration between professionals, shared consultations, therapeutic projects, team meetings and continuing education. They recognize health promotion, prevention and the clinic as important prerogatives for a resolutive PHC and, in their speech, their commitment to SUS is clear. All these elements, although admittedly effective for the systematization of work, seem to be more easily achieved when they are discussed and problematized by actors directly involved in the production of health, in the logic of co-management. Thus, dialogic reflection, based on different worldviews, instigates thinking about the practice and recognition of each segment’s role^(9)^. For all these reasons, dialogical spaces of co-management such as NCHEHU can be configured as a locus of learning and transformation, which can impact the qualitative production of health.

Finally, it should be noted that the final product of this study resulted in a “Guide for the systematization of team work”, whose development process will be addressed in other articles.

### Study limitations

Because it deals with the reality of a small municipality, which does not favor the generalization of the findings to other social contexts, although it can generate identification in spaces of similar structure. Also, low compliance of the social control segment is seen as a problem, which has been repeated in other movements of this nature.

### Contributions to nursing

Contributions to nursing reside in the insertion of NCHEHU as a possibility to carry out the Brazilian National Policies on Continuing Education in Health (PNEPS - *Política Nacional de Educação Permanente em Saúde*) and Humanization (PNH - *Política Nacional de Humanização*) at the municipal level. This is because the participation of nurses in the creation of this structure was significant. In this regard, the study contributes to strengthening co-management initiatives, valuing the participation of nursing, a profession that has been a protagonist in these processes, and giving visibility to the development of management technologies produced within the scope of PHC.

## FINAL CONSIDERATIONS

When analyzing the SWOT matrix variables perceived by research participants, it is noted that strengths and opportunities are sufficient to reverse some problems, identified as weaknesses and threats. Among these, the main point to be worked on was CHE, which in this reality emerges as a potential solution from the creation of a NCHEHU, with a significant participation of nursing.

It was evident that there are many processes that influence the systematization of work in a municipality’s health services, depending on the profile of the team and specific situations. Thus, the SWOT matrix application was considered an important strategy for (re)knowledge of local reality, making it necessary to perpetuate actions of this nature.

The representatives of care, management segments, as well as social control, perceived themselves as leaders, when identifying their role, during the planning discussion of possible actions to reverse the critical knots of everyday life, with a view to improving organization of work. It is worth highlighting the active participation of nursing as a professional category that stands out in studies of this nature, which have as their object work management in PHC.
